# Precise Determination of the Temperature Gradients in Laser-irradiated Ultrathin Magnetic Layers for the Analysis of Thermal Spin Current

**DOI:** 10.1038/s41598-018-29702-1

**Published:** 2018-07-27

**Authors:** Srivathsava Surabhi, Dong-Jun Kim, Phuoc Cao Van, Viet Dong Quoc, Jeong-Mok Kim, Sung Woo Lee, Rambabu Kuchi, Jae-Woong Lee, Soon-Gil Yoon, Jihoon Choi, Byong-Guk Park, Jong-Ryul Jeong

**Affiliations:** 1 Graduate School of Energy Science and Technology, Chungnam National University, Daejeon, 34134 South Korea; 2 Department of Materials Science and Engineering, KAIST, Daejeon, 34141 South Korea; 3 Department of Materials Science and Engineering, Chungnam National University, Daejeon, 34134 South Korea

## Abstract

We investigated the temperature distribution induced by laser irradiation of ultrathin magnetic films by applying a finite element method (FEM) to the finite difference time domain (FDTD) representation for the analysis of thermal induced spin currents. The dependency of the thermal gradient (∇*T*) of ultrathin magnetic films on material parameters, including the reflectivity and absorption coefficient were evaluated by examining optical effects, which indicates that reflectance (*R*) and the apparent absorption coefficient (α^*^) play important roles in the calculation of ∇*T* for ultrathin layers. The experimental and calculated values of *R* and α^*^ for the ultrathin magnetic layers irradiated by laser-driven heat sources estimated using the combined FDTD and FEM method are in good agreement for the amorphous CoFeB and crystalline Co layers of thicknesses ranging from 3~20 nm. Our results demonstrate that the optical parameters are crucial for the estimation of the temperature gradient induced by laser illumination for the study of thermally generated spin currents and related phenomena.

## Introduction

Recently, the spin/charge interconversion phenomena have attracted much attention from researchers. The application of thermal gradients (∇*T*) to magnetic nanostructures has opened a new era of spin current generation in the field of spintronics^1–5^. These novel spin current generation methods have enabled the discovery of the spin counterparts of the Nernst and Seebeck effects, and thus unify the spin-degree of freedom with thermoelectrics (TE). They facilitate in yielding an abundant amount of waste-heat energy-harvesting capabilities that have applications to green information communication and quantum technologies^6–8^. Various heating methods have been proposed to investigate the thermal spin currents, such as Joule^9^, Peltier^10^, microwave^11^, and laser irradiation heating^12,13^. In general, the Peltier and laser irradiation methods are widely studied because of their promising features. Peltier and Joule heating typically assume the linear temperature gradients at the interfaces of normal-metal/ferromagnetic (FM) layers; hence, they have limitations that hinder the understanding of exact nature of thermal spin current generation induced by vertical temperature differences (Δ*T*) in novel spintronic devices with thicknesses in the nanometre range^14^. Spatially resolved laser-induced studies elegantly elucidate the clear interplay between ∇*T* and the spin degree of freedom. For example, the spin Nernst magnetoresistance has been successfully demonstrated using a controllable asymmetric laser irradiation method in ferromagnet/non-magnet bilayers^15^. The laser irradiation method has employed in many spin/charge conversion studies, due to its inherent simplicity with respect to generating ∇*T* in spin TE devices. However, the precise determination of the ∇*T* distribution is yet to be resolved, as it requires both optical and thermal transport calculations in the ultrathin region.

Heat transport in low-dimensional systems, comparable to the phonon mean free path, is of key importance for a variety of applications, e.g., waste-heat harvesting devices and phonon computing^16,17^. In addition, optical parameters such as reflectivity (*r*) and absorption coefficient (*α*) can significantly vary when the optically thick film becomes ultrathin due to multiple reflections and interference effects. In the heat transfer equation, the external heat source and heat transport are defined by the optical and thermal parameters of ultrathin films correlated with the incident laser-power distribution and thermal conductivity (*κ*). Therefore, they must be determined carefully for the precise estimation of ∇*T*. The laser-induced temperature gradient in thin films was calculated using analytical model^18^ and finite element method (FEM) simulation^12^ in which the variation of thermal properties with film thickness was incorporated, however, the dependence of optical properties on film thickness was ignored.

In this paper, we propose a unique way through combining the finite difference time domain (FDTD) and a finite element method (FEM) to unravel the optical and thermal interrelated problems while calculating the ∇*T* in ultrathin layers. The laser-power distribution in ultrathin films was calculated by performing three-dimensional (3-D) full-wave simulations with appropriate boundary conditions for the precise calculation of ∇*T*. We validated the proposed method by measuring the optical parameters of amorphous and crystalline FM thin films. Our formulated approach is useful for overcoming the complexities associated with experimental procedures for obtaining the values of the effective optical parameters required for accurately estimating Δ*T* and the precise ∇*T* in ultrathin films.

## Results

**Figure 1:**
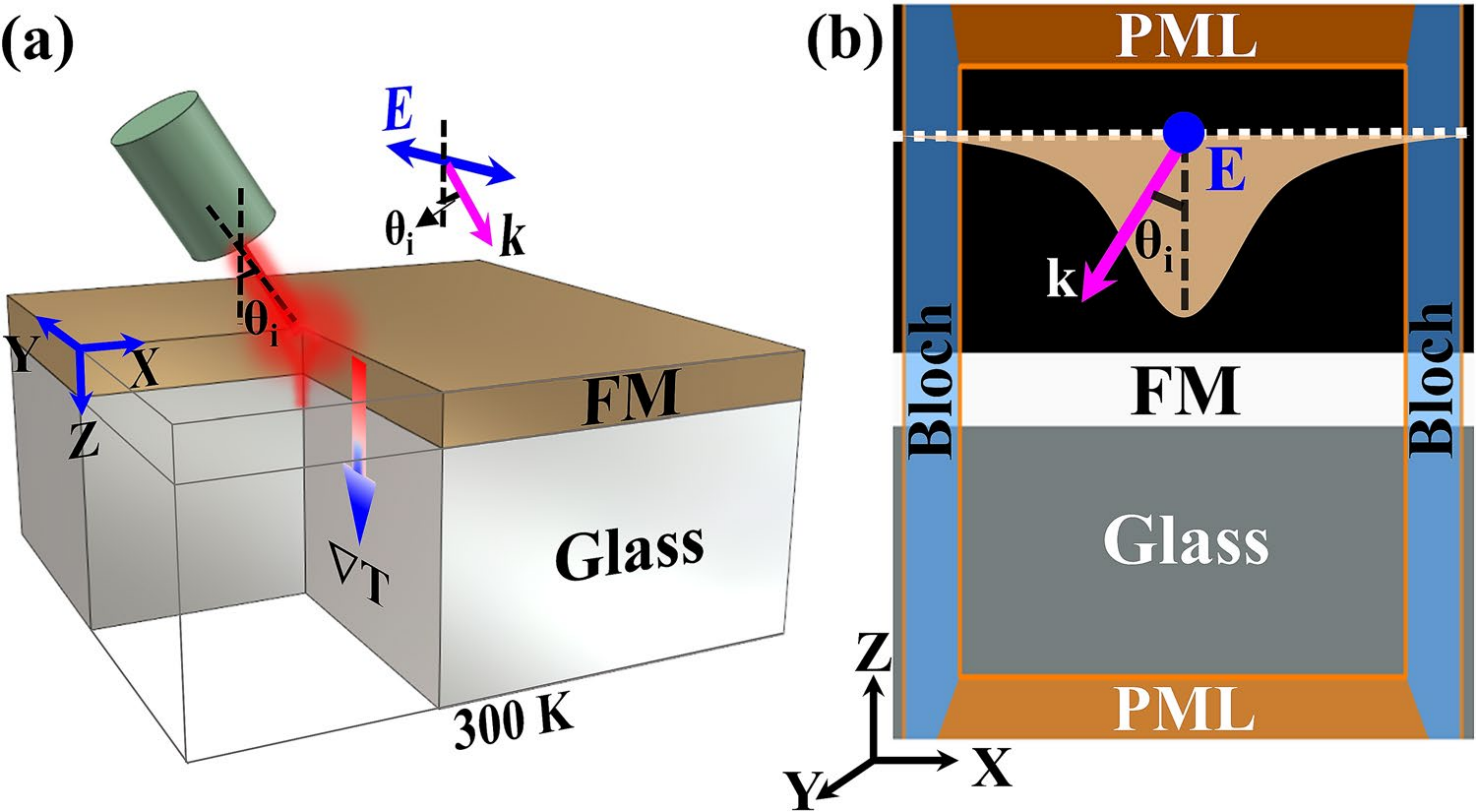
(**a**) Schematic diagram of the longitudinal orientation of the setup of the combined finite element method (FEM) and finite difference time domain (FDTD) simulations used to study the thermal gradient distribution in ferromagnetic (FM) layers under the S-polarised Gaussian laser. (**b**) The XZ plane of the actual three-dimensional (3-D) FDTD simulation environment used to estimate the optical material parameters. Perfectly matched layer (PML) boundary conditions are employed in Z-direction and Bloch boundary conditions in X, Y-directions. In here, the ‘*E*’ is electric field vector (oscillating along Y-axis), ‘*k*’ is propagation vector (along Z-axis), ‘*θ*_*i*_’ is the incident angle.

We used a longitudinal spin Seebeck effect (LSSE) geometry with the laser irradiation method shown in Fig. 1(a) as a model system for calculating ∇*T* in ultrathin films^19,20^. The ∇*T* created by laser irradiation of the FM layer, which usually has the same dimensions as the spin diffusion length, generates a spin current that can be converted into a measurable charge current by the inverse spin Hall effect. Here, the ∇*T* in the FM layer, and/or a Δ*T* across the FM/nonmagnetic interface, generates a spin current that carries angular momentum parallel to the magnetization of the FM layer^13^.

The ∇*T* and Δ*T* can be calculated by solving the Fourier equation, $\rho {C}_{p}\frac{\partial T}{\partial t}=\nabla \cdot (\kappa \nabla T)+Q$, where *ρ*, *C*_p_, *Q* are the density, specific heat capacity, and external heat source, respectively^12,21^. According to Kaiser *et al*., when the boundary conditions are properly specified, the results obtained using Fourier’s Law without modifying the bulk *κ* are essentially in exact quantitative agreement with the phonon Boltzmann equation, even in the ballistic and diffusive limits^22^. Therefore, in this study, we used the Fourier equation and FEM-based COMSOL Multiphysics software to solve the equation. In laser irradiation method, *Q* can be defined in terms of variation of laser intensity with the depth of film is given as $I(t)=(1-r)\cdot {I}_{0}\cdot {e}^{-\alpha t}$^23,24^. Here, *I*_0_, *r*, and *α* are the incident laser power, reflectivity, and absorption coefficient, respectively.

**Figure 2:**
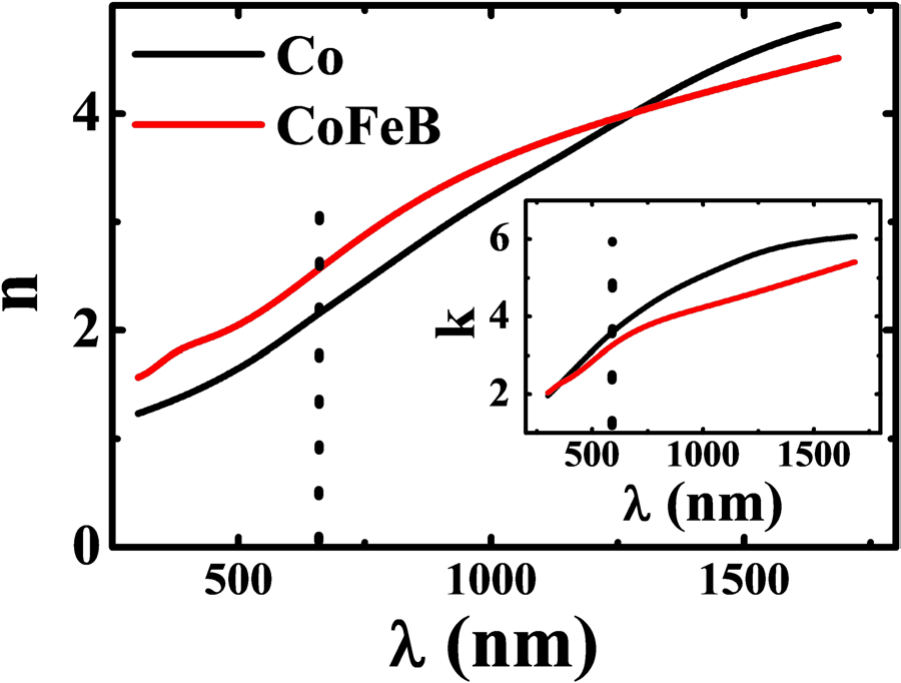
Ellipsometry measurement of the optical constants (index of refraction (*n*), extinction coefficient (*k*)) for CoFeB and Co 50-nm-thick films estimated for the spectrum between 300 to 1700 nm. The corresponding values of (*n, k*) opted for the source wavelength (660 nm; indicated by the dotted line) in this study.

Note that, in the case of ultrathin films, *r* and *α* should be replaced by the reflectance (*R*) and apparent absorption coefficient (α^*^) due to the finite thickness (*t*) effect. The parameter *α* is a thickness-independent optical constant, but the apparent electromagnetic wave absorption in the optically thin region is strongly influenced by scattering, multiple reflections and interference effects. Figure 1(b) shows the 3-D FDTD system used to calculate *R* and α^*^ for a specific film irradiated by an inclined monochromatic (*λ*_*Laser*_) Gaussian laser source. From these calculations, we estimated ${R}_{s},\,{\alpha }_{s}^{\ast }$ (where ‘*s*’ denotes simulation) by inputting the optical constants (*n, k*) of an individual 50 nm thick CoFeB and Co samples (for *λ*_*Laser*_) obtained by ellipsometry technique as shown in Fig. 2 and those of the Glass (SiO_2_) substrate^25^. Note that the optical properties of glass substrate are consistent with the literature values utilized in FDTD simulation (Supplementary Note 1). To validate our calculation method, we prepared both amorphous “CoFeB” and crystalline “Co” FM ultrathin films (t = 3~20 nm) on transparent glass substrates and measured ${R}_{m},\,{\alpha }_{m}^{\ast }$ (where ‘*m*’ denotes measurement) for *λ*_*Laser*_. Thereafter, we calculated the Δ*T* and ∇*T* profiles of corresponding FM films by incorporating these ${R}_{s},\,{\alpha }_{s}^{\ast }$ and ${R}_{m},\,{\alpha }_{m}^{\ast }$ values in isotropic transient heat conduction study and compared the results.

### Investigation of temperature gradient profiles in CoFeB ultrathin layers

**Figure 3:**
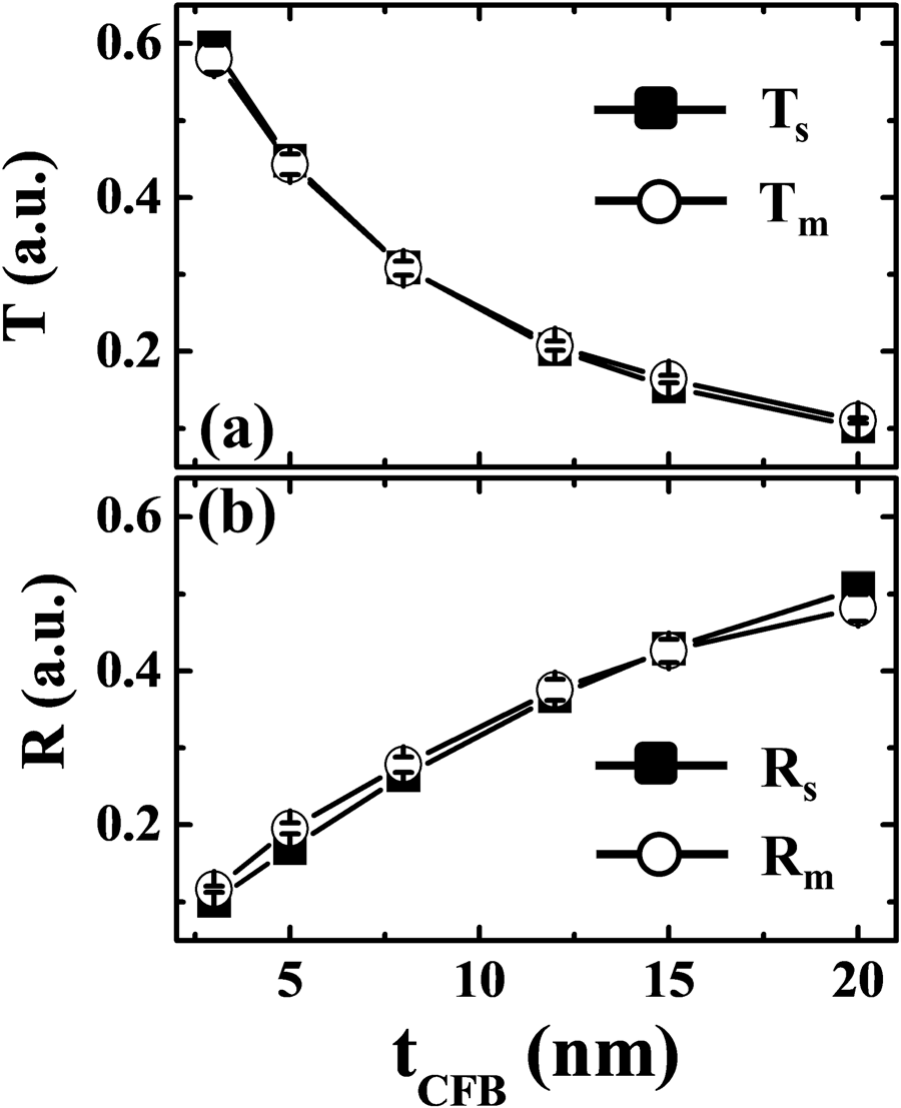
(**a**) Transmittance (*T*_*s*_, *T*_*m*_) and (**b**) reflectance (*R*_*s*_, *R*_*m*_) of CoFeB films as a function of thickness (*t*_*CFB*_: 1–20 nm). The suffixes ‘*s*’ and ‘*m*’ stand for simulated and experimentally measured values, respectively.

To prevent structural transition artifacts in the ultrathin regime, we initially considered the amorphous CoFeB films (Supplementary Note 2). At first, we obtained the transmittance (*T*) and *R* as a function of the film thickness (*t*_*CFB*_) using FDTD simulation. For this calculation, we used the optical constants (*n* = 2.57*, k* = 3.51) for *λ*_*Laser*_ of a 50-nm-thick CoFeB film (Fig. 2). Figure 3 shows the *T*_*s*_ and *R*_*s*_ values calculated when changing the film thickness from 3 to 20 nm. For comparison, we plotted the *T*_*m*_ and *R*_*m*_ values measured by the power meter together. In this paper, the suffixes ‘*s’* and ‘*m*’ denote simulated and measured values, respectively. As the film thickness decreased, the transmission was enhanced and the reflectance diminished due to the effective reduction of the optical path length^26^. Note that the simulated and measured values were in good agreement. The error bars indicate the deviations associated with the numerous experimental measurements of (*T*_m_*, R*_m_).

**Figure 4:**
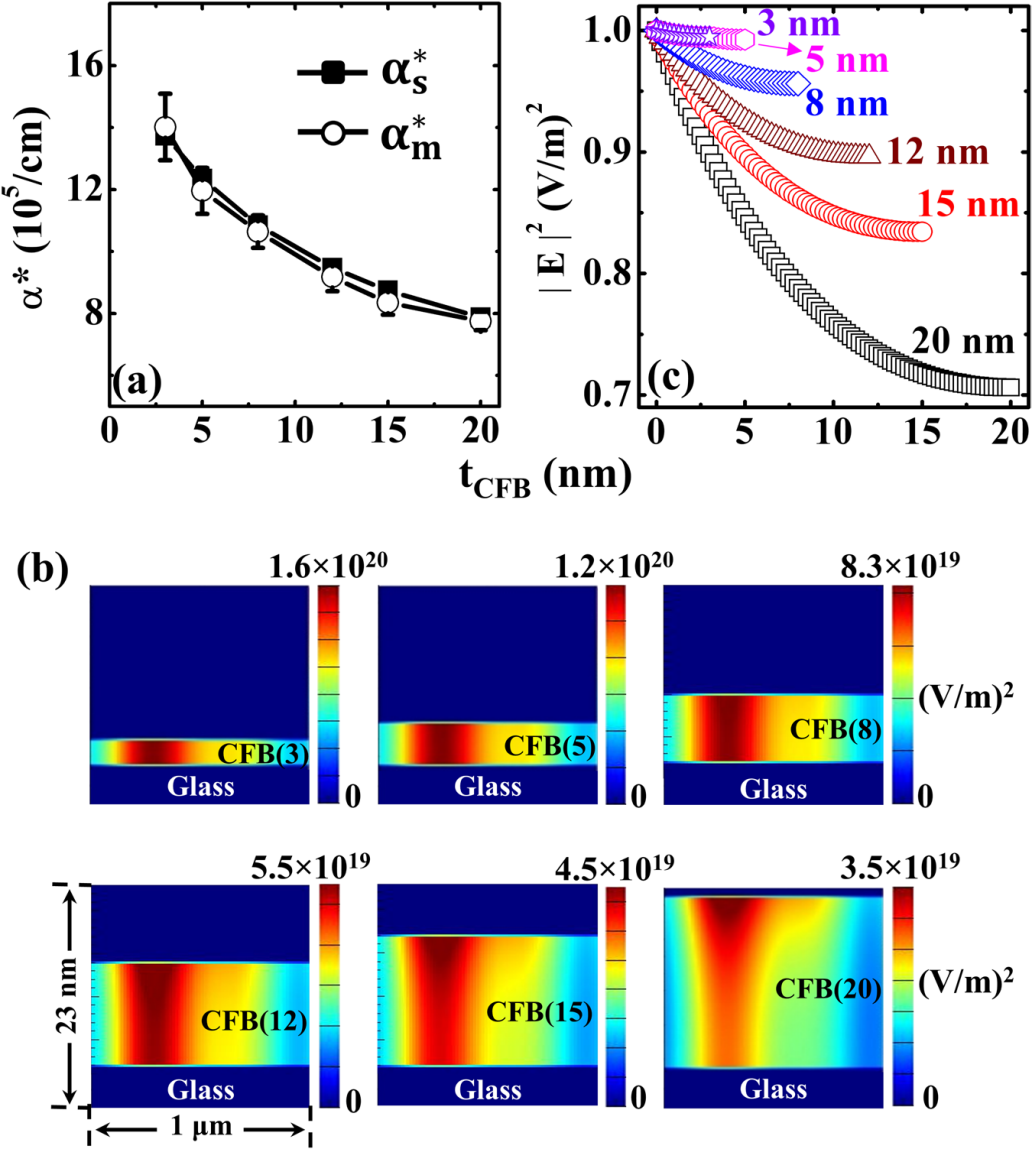
(**a**) Variation between the simulated and measured apparent absorption coefficients (α^*^) of CoFeB thin films (*t*_*CFB*_: 1–20 nm). (**b**) The Gaussian distribution of electric field intensity $({|E|}^{2})$ absorption in the *t*_*CFB*_ layers. (**c**) Numerically calculated normalised ${|E|}^{2}$ absorption as a function of *t*_*CFB*_ at the point of laser incidence.

Figure 4(a) shows the variation of α^*^ as a function of *t*_*CFB*_, which characterizes the dissipation of the laser power irradiating the CoFeB ultrathin films. ${\alpha }_{s,\,m}^{\ast }$ was calculated based on *R*_s_, *T*_s_ and *R*_m_, *T*_m_ values using the relation, ${\alpha }^{\ast }=-\,\frac{1}{t}\,\mathrm{ln}\,\frac{T}{1-R}$^23^. The simulated and experimental α^*^ values have greatly agreed with each other in the ultrathin regime. The enhanced transmission led α^*^ tends to increase with the decrement of *t*_*CFB*_, indicating a qualitative enhancement of absorption ability within the layer as the incident radiation passed through it. Thickness-dependent α^*^ of the ultrathin films clearly demonstrates the importance of carefully inputting the optical parameters when using the laser irradiation method.

Thereafter, we emphasize to account the expected interference and multiple reflection effects in the ultrathin films by the full wave simulation. To justify this assertion, the contour plots of 3-D FDTD simulated electric field intensity $({|E|}^{2})$ absorption profiles across *t*_*CFB*_ for *λ*_*Laser*_ were presented in Fig. 4(b). As *t*_*CFB*_ increases, we can see the Gaussian distribution of the incident laser power (in the 20-nm-thick CoFeB film) abide by the skin depth. We note that the decay of the $\,{|{\rm{E}}|}^{2}$ is prominently observed within the films due to the poor absorption of the glass substrate. The resulted absorption profile (in terms of α^*^) within the FM film shown in Fig. 4(b) accounts for the optical effects that occur in ultrathin films. Note that ${\alpha }_{s}^{\ast }$ and ${\alpha }_{m}^{\ast }$ are in strong agreement with respect to the film thickness. To elucidate the increase of α^*^, we quantified the electric (*E*)-field decay within the films and plotted the variation of ${|E|}^{2}$ as a function of *t*_*CFB*_ at the point of incidence in Fig. 4(c). Here, the ${|E|}^{2}$ was normalised by the value of the respective field intensity obtained from the point of incidence on the top surface of the films. Hence, the influence of multiple reflections and interference effects on the incident radiation absorption in thin films within the limits of the skin depth, were accounted for.

**Figure 5:**
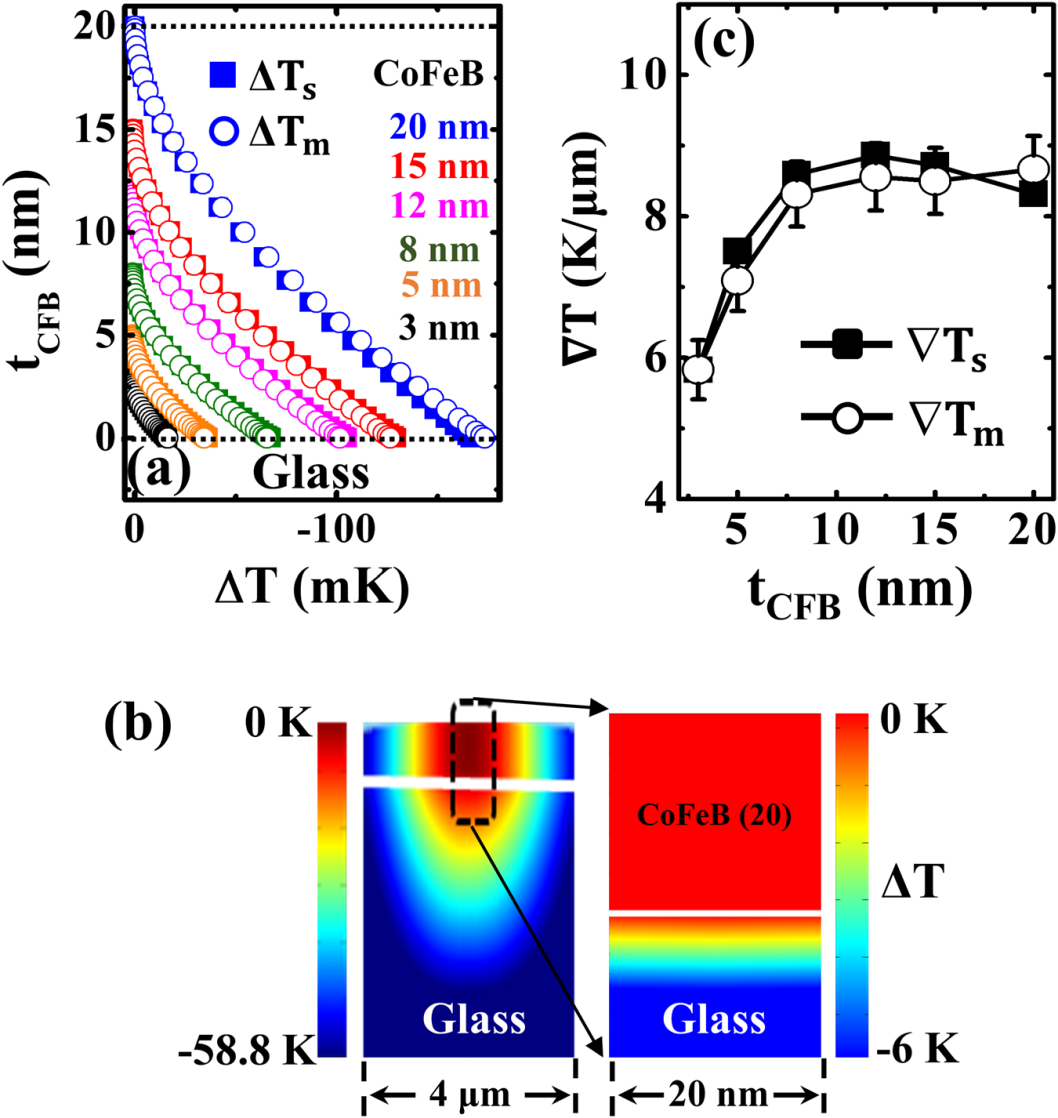
FEM simulation results: (**a**) Temperature profile (Δ*T*_*s, m*_) (mK) distribution across the *t*_*CFB*_ films. (**b**) Side view of the temperature profile map in CoFeB/Glass around the area of 4-μm irradiated by the laser beam (left) and nearby the 20-nm area (right) (**c**) Normalised thermal gradient (∇*T*_*s, m*_) variation as a function of *t*_*CFB*_.

We then performed the heat transfer simulations to obtain the Δ*T* distributions in the FM layer by applying the material parameters as listed in Table 1^19,27,28^ in the Fourier equation^21^. Figure 5(a) shows the calculated profiles of the Δ*T*_*s, m*_ (mK) at the center of the laser spot along the (CoFeB) film normal based on simulated and measured parameters. Δ*T*_*CFB*_ is gradually diminishing with decreasing *t*_*CFB*_, and both experimental and numerical results are in excellent agreement. The Δ*T* between the top and bottom surfaces of the 20-nm CoFeB layer, calculated from the simulated and measured parameters, were 166 and 173 mK, respectively. The amount of heat absorbed by the deposited layers increases with respect to the thickness of films. Figure 5(b) is the side view of the temperature distribution for the 20-nm-thick CoFeB film around the focused laser spot area. The precise calculation of ∇*T* across the sample is used to implement in spin-charge conversion experiments for the analysis of thermodynamic state at the sample interfaces. The ∇*T* is obtained by normalising the Δ*T*_*CFB*_ with the respective *t*_*CFB*_ as illustrated in Fig. 5(c).

**Table 1:** Material parameters used in the calculations of the temperature distributions.

Material	*ρ*_m_ (10^3^ kg∙m^−3^)	*C*_p_ (J∙kg^−1^∙K^−1^)	*κ* (W∙m^−1^∙K^−1^)	*α* (10^5^ cm^−1^)	*R* (a.u.)	*r* (a.u.)
CoFeB	8.22	440	86.7	Fig. 4(a)^*^	Fig. 3(b)^*^	—
Co	8.90	421	100	Fig. 6(c)^*^	Fig. 6(b)^*^	—
SiO_2_	2.20	1052	1.4	10^−11^	—	0.045
Si	2.33	700	150	0.0102	—	0.33

*ρ*_m_, *C*_p_, *κ*, *α, R, and r* are, respectively, the density, specific heat, thermal conductivity, absorption coefficient, reflectance, and reflectivity. ^*^In this case, we used both the simulated and measured values.

### Investigation of temperature gradient profiles in Co ultrathin films

**Figure 6:**
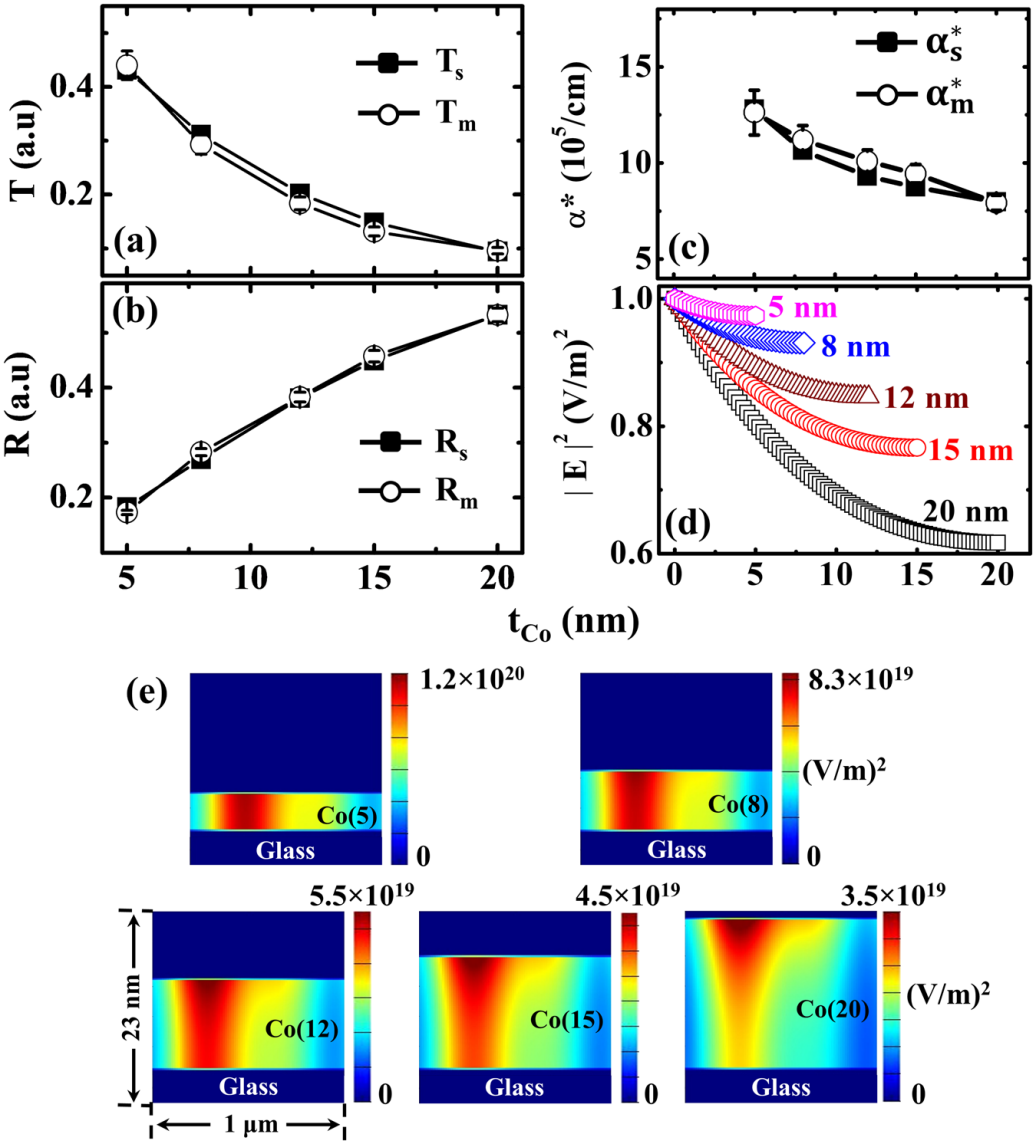
Summary of the results for crystalline Co ultrathin magnetic layers of thickness (*t*_*Co*_) range between 5–20 nm. Variation of (**a**) transmittance (*T*_*s*_, *T*_*m*_) and (**b**) reflectance (*R*_*s*_, *R*_*m*_). (**c**) Variation of simulated and measured apparent absorption coefficient (α^*^). (**d**) Numerically calculated normalised ${|E|}^{2}$ absorption in *t*_*Co*_ layers at the point of laser incidence. (**e**) The Gaussian distribution of electric field intensity $({|E|}^{2})$ absorption in the *t*_*Co*_ layers.

We further confirmed the validity of our method by including crystalline Co ultrathin films (*t*_*Co*_ = 5~20 nm) in our study (Supplementary Note 2). It is well known that the structure of ultrathin Co film is metastable and exhibits a wide range of thickness-dependent structural transitions that depend on the under layer and capping layer^29,30^. This implies that difficulties may arise when conducting simulation-based estimations of *R* and α^*^. We calculated (*T*_*s*_*, R*_*s*_) for *t*_*Co*_ by inserting the optical constants of 50-nm-thick Co layer for *λ*_*Laser*_ (*n* = 2.16, *k* = 3.91 from Fig. 2) in FDTD optical simulation. The calculated and measured values are plotted together in Fig. 6(a,b), respectively. We note that the grain size of the Co is less than 10 nm, which has a negligible effect on the results of the FDTD simulation (Supplementary Note 2). Thereafter, ${\alpha }_{s,\,m}^{\ast }$ is plotted in Fig. 6(c), and all the results were in good agreement when the experimental error was taken into account. We demonstrated the enhanced light absorption in ultrathin magnetic films by plotting the numerically obtained normalised ${|E|}^{2}$ absorption profiles across *t*_*Co*_, as shown in Fig. 6(d). Similar to the Figs 4(b), 6(e) shows the Gaussian distribution of incident laser power across the ‘*t*_*Co*_’ through the contour plots of ${|E|}^{2}$.

**Figure 7:**
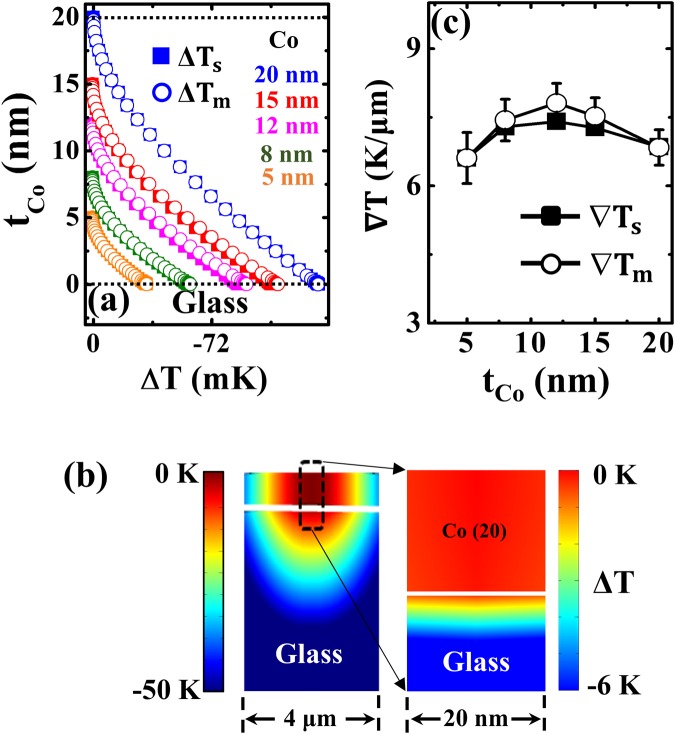
FEM simulation results: (**a**) Temperature profile (Δ*T*_*s, m*_) (mK) distribution across the *t*_*Co*_ films. (**b**) Side view of the temperature profile map in Co/Glass around the area of 4-μm irradiated by the laser beam (left) and nearby the 20-nm area (right) (**c**) Normalised thermal gradient (∇*T*_*s, m*_) variation as a function of *t*_*Co*_.

Figure 7(a) shows the variation in the Δ*T*_*s, m*_ (mK) profiles across the (*t*_*Co*_) films of 3-D heat transfer simulations using parameters in Table 1. Figure 7(b) shows the side view of the temperature profile distribution of the 20-nm-thick Co layer. The Δ*T* between the top and bottom surfaces of the 20-nm Co layer calculated using the simulated and measured parameters were 137.08 and 136.74 mK, respectively. Figure 7(c) depicts the normalised ∇*T*_*s*_, _*m*_ (K/μm) variation as a function of corresponding *t*_*Co*_. The ∇*T* increases with *t*_*Co*_ and tends to slightly decline over 15-nm, which is because the intensity of incident laser radiation is exponentially decayed as the thickness is increased and becomes negligible after a penetration depth by means of absorption. Therefore, we can confirm that our combined formulation can be used to successfully determine Δ*T*, ∇*T* distributions in ultrathin FM films, yielding an excellent agreement between temperature profiles based on simulated and experimentally calculated optical parameters for both CoFeB and Co ultrathin films by accounting the optical effects in ultrathin films.

## Discussion

Optical effects present an inherent challenge regarding the evaluation of the enhanced transmission-driven apparent absorption coefficient when using laser irradiation on ultrathin films to study thermally induced spin currents. This is the key parameter for calculating the amount of heat dissipation from the incident power. We can confirm this by the contour plots of ${|E|}^{2}$ absorption profiles within the deposited layer. The Gaussian distribution of incident energy in the ultrathin FM layer is clearly observable provided the thickness of the FM layer surpasses the skin depth. This ensures that the optical effects need to be accounted in the ultrathin FM layer itself and does it by the formulated calculation. The inherent experimental limitations that affect the calculation of the temperature distribution could be resolved by this proposed study. The Δ*T* profiles calculated across the top and bottom surfaces of the deposited layers using the experimental and simulated optical material parameters agreed well. The side view of the Δ*T* colour maps, and ∇*T* variation as a function of thickness for both amorphous (CoFeB) and crystalline (Co) structures are validating our investigation for the precise calculation of thermal gradients for spin-charge interconversion studies.

In summary, we have investigated the effect of laser irradiation on the temperature profile distributions of amorphous CoFeB and crystalline Co ultrathin FM films ranging from 3–20 nm by combining FDTD and FEM techniques. In ultrathin dimensions, the material parameters (R, α^*^) suffering from optical effects differ from their respective bulk values. Hence, we systematically investigated these parameters and explored their impact on temperature distributions in ultrathin films. Our proposed method yields the precise calculation of thermal gradients across the deposited FM layer and overcomes the limitations of experimental approaches in determining it. The precise determination of ∇*T* across the FM layer is very important when investigating thermally induced spin current generation in various spintronic devices.

## Methods

### Sample fabrication

We fabricated single-layer structures of amorphous Co_32_Fe_48_B_20_ (*t*_*CFB*_ = 3–20 nm) and crystalline Co (*t*_*Co*_ = 5–20 nm) samples by magnetron sputtering onto a commercial glass substrate of “Corning eagle XG” with a base pressure of 3 × 10^−8^ Torr, a working pressure of 3 mTorr, and a sputtering power of 30 W. An individual 50-nm thick CoFeB, Co samples are used for ellipsometry measurements to estimate the optical constants. Optical characterizations were carried out for the source wavelength range of 300~1700 nm, from which we opted the values of (*n, k*) for the *λ*_*Laser*_ (660 nm) of power 55.3 mW. *R*_*m*_, *T*_*m*_ were estimated using the UV-Visible power meter in order to calculate the thermal gradients across the films using FEM technique.

### Finite difference time domain method simulations

A monochromatic S-polarised Gaussian-shaped laser with *λ*_*Laser*_ = 660 nm was obliquely incident (θ = 32.5°, along with the Z-axis) on FM layer. The incident power was distributed across the films in Gaussian form, as shown in Fig. 1(b). Bloch boundary conditions were applied along the *X, Y-*axes, and perfectly matched layer (PML) boundary conditions were employed parallel to the source direction (*Z*-axis). ${R}_{s},\,{\alpha }_{s}^{\ast }$ were estimated using the bulk optical constants (*n, k*) in these calculations of FM layers measured in Fig. 2. We obtained the Δ*T* profile and in turn ∇*T* for the corresponding FM layer by incorporating the values ${R}_{s},\,{\alpha }_{s}^{\ast }$ in *I*(*t*) in FEM.

### Finite element method simulations

In the FEM simulations, we modelled the laser beam as a continuous 55-mW Gaussian beam with a power density distribution, ${\rm{y}}({\rm{x}})=\frac{1}{\sigma \sqrt{2\pi }}{e}^{\frac{-{(x-{x}_{0})}^{2}}{2{\sigma }^{2}}}$ and total beam width of ~3σ (equivalent to 5 μm), as shown in Fig. 1(a). This is consistent with the laser experimental condition. The effect of laser spot size on temperature distribution in CoFeB 20 nm layer is described in Supplementary Note 3. In the figure, *x*_0_ is the location (mean) on the sample irradiated by the beam, *x* is an input variable, and σ is the standard deviation. In this case, we applied open boundary conditions to all external surfaces since they are not insulated in this experiment. It allows the heat can flow out/in across all the surfaces, which only depends on the external temperature of the sample. The ambient temperature at the surface was fixed at 300 K for all simulations. We evaluated the temperature profiles of samples 400 × 400 μm^2^ of varying thicknesses under these conditions.

### Data availability

The data that support the findings of this study is available from the corresponding authors on reasonable request.

## Electronic supplementary material

Supplementary Information
