# Supplementary material for: Precise Determination of the Temperature Gradients in Laser-irradiated Ultrathin Magnetic Layers for the Analysis of Thermal Spin Current
Source: Sci Rep. 2018 Jul 27;8:11337. doi: 10.1038/s41598-018-29702-1 (PMC6063919; doi:10.1038/s41598-018-29702-1)
Supplement: Supplementary file 1 — Supplementary Information [file 41598_2018_29702_MOESM1_ESM.docx]

Supplementary Information

**Precise Determination of the Temperature Gradients in Laser-irradiated Ultrathin Magnetic Layers for the Analysis of Thermal Spin Current**

Srivathsava Surabhi^1†^, Dong-Jun Kim^2†^, Phuoc Cao Van^3^, Viet Dong Quoc^3^, Jeong-Mok Kim^2^, Sung Woo Lee^3^, Rambabu Kuchi^3^, Jae-Woong Lee^3^, Soon-Gil Yoon^3^, Jihoon Choi^3^, Byong-Guk Park^2^*, Jong-Ryul Jeong^1,3^*

^1^Graduate School of Energy Science and Technology, Chungnam National University, Daejeon 34134, South Korea.

^2^Department of Materials Science and Engineering, KAIST, Daejeon 34141, South Korea.

^3^Department of Materials Science and Engineering, Chungnam National University, Daejeon 34134, South Korea.

*Correspondence and requests for materials should be addressed to J.-R.J. (email: jrjeong@cnu.ac.kr), B.-G.P. (email: bgpark@kaist.ac.kr)

†These authors contributed equally to this work.

**Table of contents**

**Note 1. Optical constants of Glass substrate of experimental and FDTD simulation utilized for the laser source wavelength**

**Note 2. X-ray diffraction (XRD) analysis of the CoFeB, Co ultrathin films**

**Note 3. Effect of laser spot size on the temperature distribution in CoFeB 20 nm ultrathin films**

**Note 1. Optical constants of Glass substrate of experimental and FDTD simulation utilized for the laser source wavelength**

The SiO_2_ in FDTD simulation refers to the glass substrate used in experiments. In the simulation, we used the optical properties of SiO_2_ (Glass) from literature [S1]. We experimentally measured (n_m_, k_m_) of the glass substrate (Corning eagle XG), which are consistent with the simulation ones (n_s_, k_s_) as shown in Fig. S1. The dotted line indicates to 660 nm that is opted for the FDTD simulation.


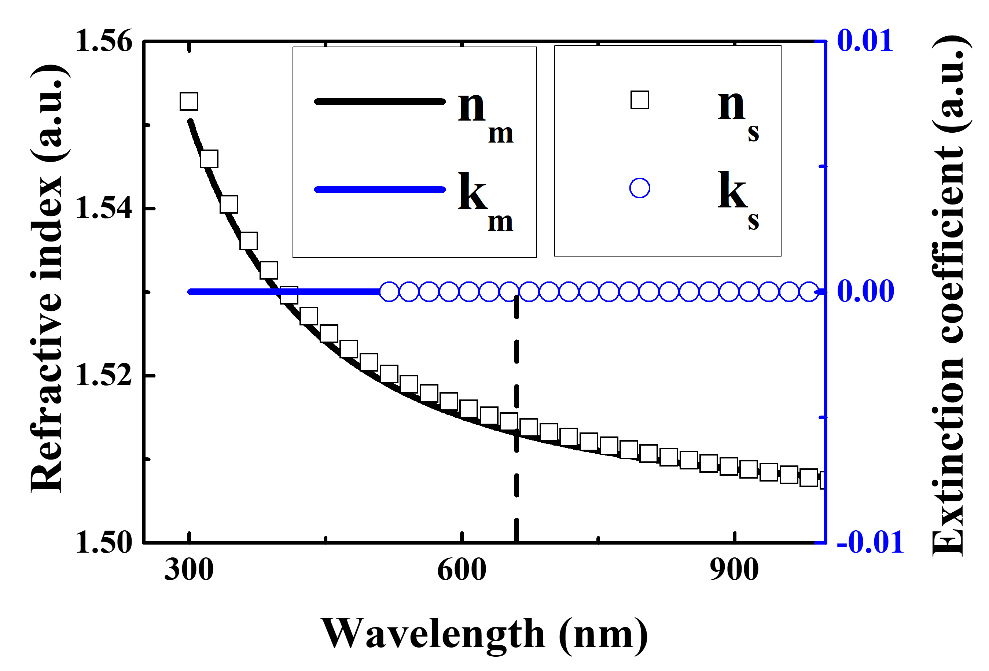


**Figure S1:** Optical constants (n, k) of the Corning Eagle XG glass substrate (m-experimental), and SiO_2_ (Glass) (s-simulation).

**Note 2. X-ray diffraction (XRD) analysis of the CoFeB, Co ultrathin films**

We measured the X-ray diffraction (XRD; model D/MAX-2500; RIGAKU, Cu-X-ray source) of the CoFeB and Co film (5 nm, 20 nm, and 50 nm). This clearly depicts the amorphous nature of CoFeB films [Fig. S2 (a)] and a crystalline Co of hexagonal close packing (HCP) structure [Fig. S2 (b)]. The latter is confirmed by the comparison of the X-ray diffraction data with the reference data of HCP Co [PDF#00-005-0727], which is shown in Fig. S2 (c). The diffraction peaks show a preferential growth plane of (002) for the Co sample thicker than 20 nm, while those are not clearly distinguished for the 5 nm film. Therefore, we think that there is no structural change as a function of thickness.

Moreover, we extracted a grain size of ~6.5 nm (~10 nm) for Co 20 nm (50 nm) film using the Scherrer’s equation and the XRD (002) peaks as shown in Fig. S2 (c). To check the effect of the grain size on optical material parameters, we performed FDTD simulation with and without considering the grain size of 10 nm. The differences in the calculated transmittance and reflectance data are less than 0.1%, demonstrating that there is a negligible effect of grain size (10 nm) on the result of our study.


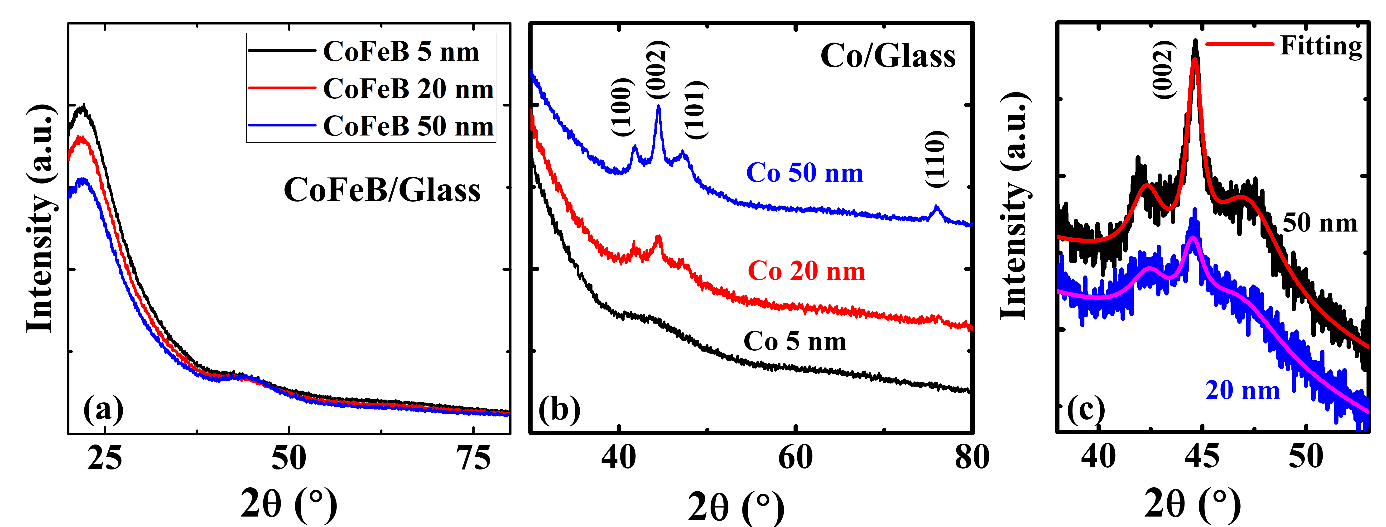


**Figure S2**. XRD analysis of 5, 20, 50 nm thick films of (a) CoFeB, (b) Co deposited on Glass. (c) Fitted profiles of 2θ scan for Co 20, 50 nm films.

**Note 3. Effect of laser spot size on the temperature distribution in CoFeB 20 nm film**

It is expected that when the size of the laser increases while maintaining the same power, the lateral temperature distribution is increased, resulting in a decrement in the temperature difference (ΔT) across the film. We performed additional simulations with different laser spot size of 10 μm, confirming the reduction in ΔT for a larger laser spot as compared to that using a laser spot size of 10 μm (Fig. S3).


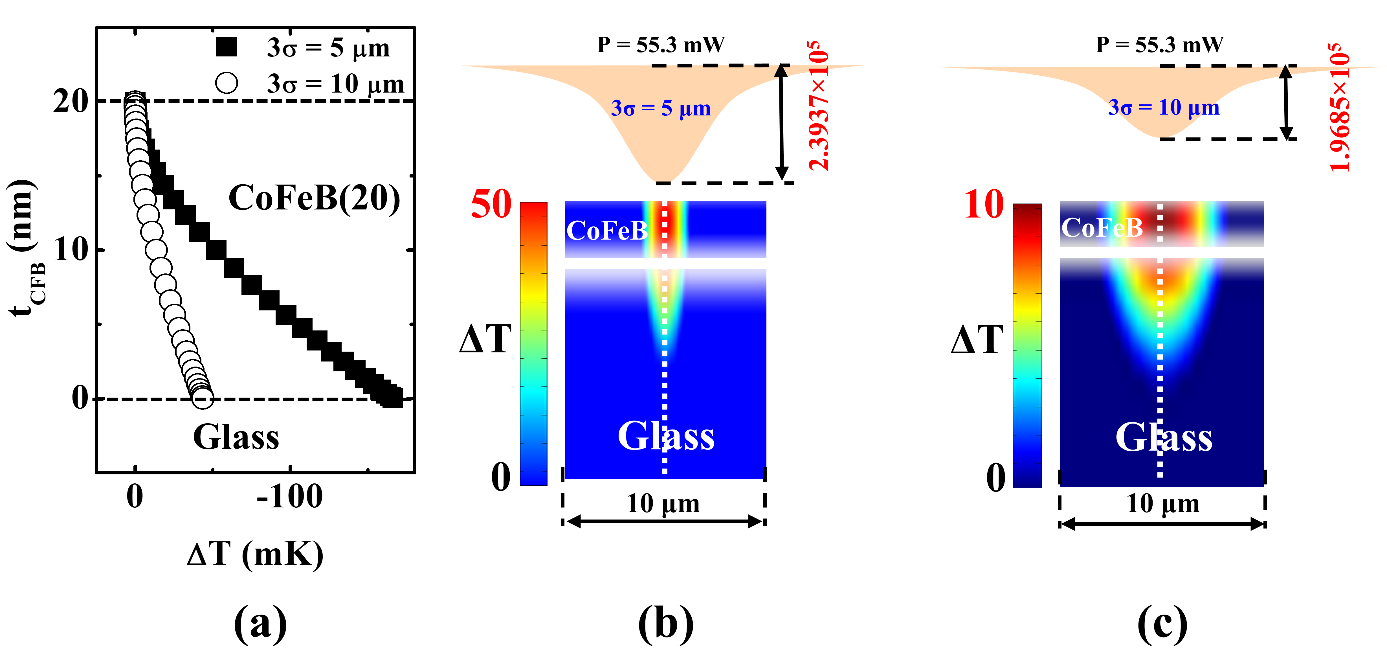


**Figure S3**. (a) Temperature difference profiles for the laser 3σ of 5, 10 μm. Side view of the temperature distributions for the 3σ of (b) 5 μm (c) 10 μm.

Reference

[S1] Palik, E. D. *Handbook of Optical Constants of Solids*. **I-III,** (1985).
